# Kv7 channel trafficking by the microtubule network in vascular smooth muscle

**DOI:** 10.1111/apha.13692

**Published:** 2021-06-04

**Authors:** Thomas A. Jepps

**Affiliations:** ^1^ Vascular Biology Group Department of Biomedical Sciences University of Copenhagen Copenhagen N Denmark

**Keywords:** caveolae, dynein, KCNQ, Kv7, microtubules, vascular

## Abstract

In arterial smooth muscle cells, changes in availability of integral membrane proteins influence the regulation of blood flow and blood pressure, which is critical for human health. However, the mechanisms that coordinate the trafficking and membrane expression of specific receptors and ion channels in vascular smooth muscle are poorly understood. In the vasculature, very little is known about microtubules, which form a road network upon which proteins can be transported to and from the cell membrane. This review article summarizes the impact of the microtubule network on arterial contractility, highlighting the importance of the network, with an emphasis on our recent findings regarding the trafficking of the voltage‐dependent Kv7 channels.

## 1 POTASSIUM CHANNELS REGULATE ARTERIAL TONE

The resting membrane potential of smooth muscle cells is dictated by the dominant potassium (K^+^) conductance. The opening of K^+^ channels results in K^+^ efflux from the cell and membrane hyperpolarization, which opposes membrane depolarization and reduces the open probability of voltage‐dependent calcium channels, thereby attenuating smooth muscle cell contraction.^1^ Several different K^+^ channels are expressed in vascular smooth muscle cells, including voltage‐dependent (Kv), ATP‐sensitive, inward‐rectifying, and large‐conductance, calcium‐activated (BK_Ca_) channels.^2^ The contribution of each of these channels to the resting membrane potential of vascular smooth muscle, as well as arterial and myogenic tone, has been described; however, the impact of each of these channels is often dependent on the arterial preparation, technique used to measure arterial responses and pharmacological intervention.^2^ Depending on the blood vessel and experimental approach, Kv channels and their ancillary subunits can contribute to the resting membrane potential, often together with other K^+^ channel subtypes, for example, BK_Ca_ channels.^2^, ^3^, ^4^, ^5^, ^6^, ^7^, ^8^, ^9^, ^10^, ^11^ Importantly, the equilibrium potential of K^+^ in arterial smooth muscle is estimated to be ~80 mV,^12^ thus a K^+^ conductance does not completely dominate the resting membrane potential because of various depolarizing influences, such as cation channels, and a chloride conductance.^13^ Within the Kv channel superfamily (Kv1‐Kv12), Kv1.2, Kv1.5, Kv2.1, Kv7.4 and Kv7.5 channels have been identified consistently in an array of different blood vessels from rodents, rabbits, dogs, swine and humans.^2^, ^4^, ^5^, ^14^, ^15^

## 2 Kv7 CHANNELS IN VASCULAR SMOOTH MUSCLE

The Kv channels form the largest and most diverse family among human K^+^ channels with 12 known subfamilies (Kv1‐Kv12).^16^ The K^+^‐conducting pore of the Kv channel is formed from four α‐subunits (each between 55 and 77 kDa), each consisting of six transmembrane domains (S1‐S6).^17^ The S5 and S6 transmembrane domains of all four monomers collectively arrange to form a single ion‐conducting pore in the centre of the structure.^17^ Attached to S1 and S6 are the intracellular N‐ and C‐termini, respectively, which are involved in tetrameric channel assembly, channel gating and trafficking, as well as providing intracellular binding sites for multiple signalling molecules. S1‐S4 of each α‐subunit arrange at the periphery of the pore to form distinct voltage sensors.^18^, ^19^, ^20^ The S4 domain is the main transmembrane voltage‐sensing component, containing a motif of four to six basic amino acid residues creating a positively charged surface, which is responsible for the sensitivity of the channel to changes in the membrane potential.^21^, ^22^

Kv7 channels (Kv7.1‐7.5) are slowly activating, non‐inactivating, voltage‐dependent K^+^ channels.^23^ Importantly, these channels are active at more negative membrane potentials compared to other Kv channels making them perfect candidates for the K^+^ current controlling the resting membrane potential of vascular smooth muscle cells. Over the past 20 years, a key role in vascular (and non‐vascular) smooth muscle has been established, with Kv7.4 and Kv7.5 channels predominating,^24^, ^25^, ^26^, ^27^, ^28^, ^29^, ^30^, ^31^, ^32^ which are likely to be regulated by the ancillary subunit KCNE4.^5^, ^33^, ^34^ Several reviews have summarized the evidence supporting an important role for Kv7.4 and Kv7.5 channels in vascular smooth muscle.^23^, ^35^, ^36^, ^37^

Like many voltage‐gated channels, Kv7 channels also respond to a variety of different ligands.^38^, ^39^ Thus, as well as controlling the resting membrane potential of arterial smooth muscle cells, Kv7.4 and Kv7.5 channels are downstream targets in several signalling pathways, including secondary messengers cAMP and cGMP.^26^, ^40^, ^41^, ^42^, ^43^, ^44^, ^45^, ^46^ For example, agonist binding to Gs protein‐coupled receptors, including β adrenergic, adenosine and calcitonin gene‐related peptide receptors leads to a downstream activation of vascular Kv7 channels, which contributes to the elicited vasorelaxation (for review see Ref.^38^). Moreover, Kv7 currents are decreased upon protein kinase C (PKC) activation in mesenteric and cerebral artery myocytes because of PKC phosphorylation of the Kv7.5 channels.^25^, ^47^ In addition, Kv7 channels contribute to the myogenic response in cerebral, basilar and gracilis arteries.^26^, ^31^, ^48^, ^49^ Pharmacological activation of the Kv7 channels suppressed myogenic tone development at pressures >20 mmHg in cerebral arteries mounted in a pressure myograph, whereas blockade of Kv7 channels enhanced the myogenic response.^48^

In arteries from hypertensive animals, Kv7 function is attenuated across different vascular beds, which is concomitant with reduced Kv7.4 protein expression.^40^, ^41^, ^50^, ^51^, ^52^, ^53^ Importantly, because of the role of Kv7 channels in basal smooth muscle control and relaxant response to receptor agonists, decreased Kv7 channel function will contribute to the progressive increase in arterial tone and resistance of vasodilators manifest in hypertension. Hence, the decreased Kv7 channel function in arteries from hypertensive animals is reflected in impaired relaxations to certain Gs protein‐coupled receptor agonists, such as isoprenaline, which activates β adrenoceptors.^54^

Ion channels and receptors, including Kv7 channels, must be trafficked accurately and ultimately be internalized and degraded at the appropriate times to ensure normal signalling pathways and membrane potentials are maintained.^55^, ^56^ In the case of Kv7 channels, the removal and insertion of new channels needs to be timed accurately to maintain their total cellular conductance. If this timing were not accurate, a decreased number of channels would lead to reduced conductance and change in arterial tone. In addition, Kv7 channels also behave as ligand‐gated channels and are downstream targets of multiple signalling pathways. In order for intracellular secondary messengers to regulate channel activity, it is important the channel is accurately trafficked to certain cell membrane domains, for example, caveolae and lipid rafts, to maintain close proximity with the specific secondary messengers. Although key roles for Kv7.4 and Kv7.5 channels in vascular smooth muscle have been established, as well as a role in hypertension, the mechanisms regulating the trafficking and localization of the channels to and from the smooth muscle cell membrane are poorly understood.

## 3 THE MICROTUBULE NETWORK

**FIGURE 1:**
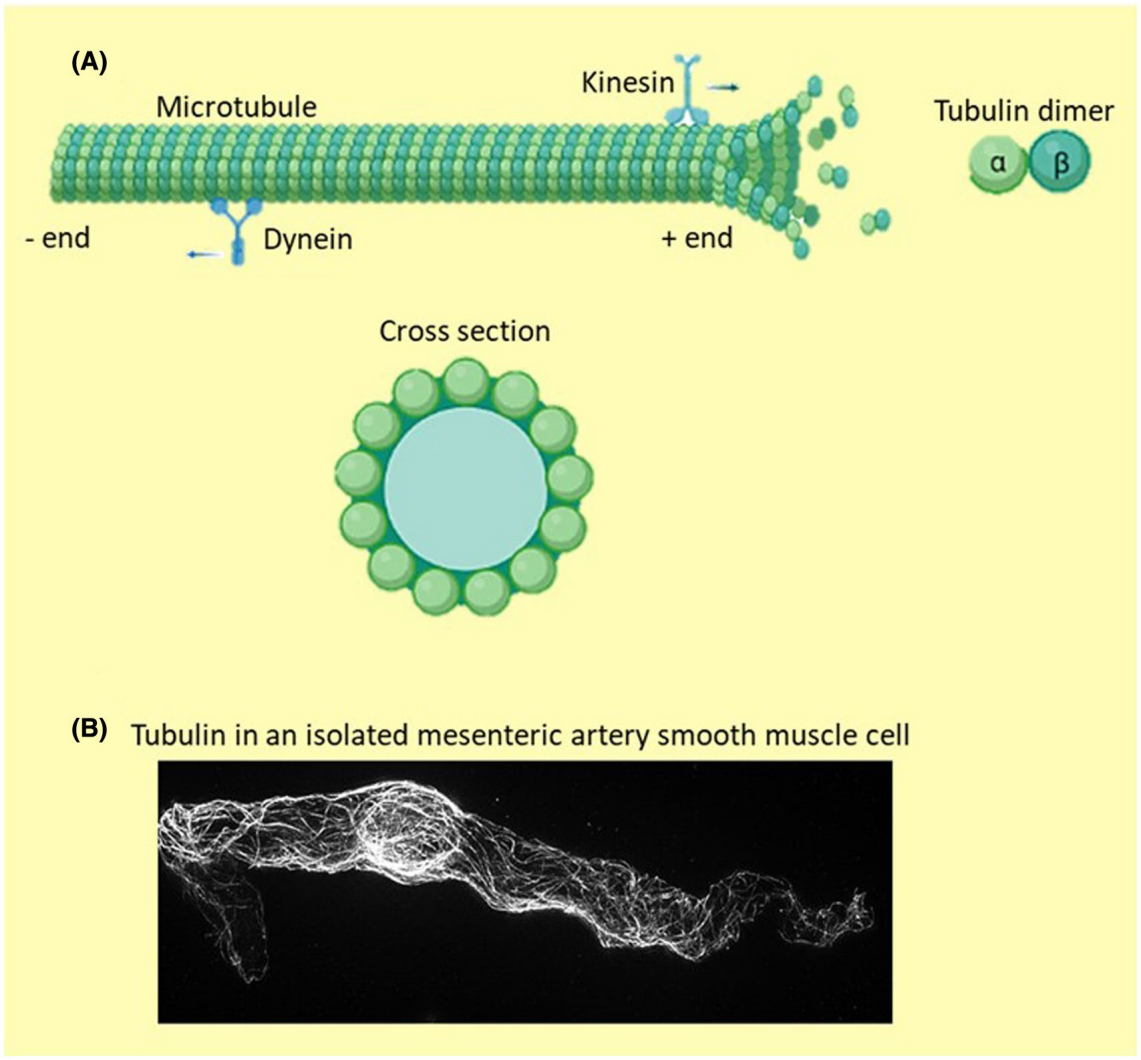
A, Illustration of a microtubule formed by dimers of α and β tubulin attaching to the polymerizing microtubule plus end. Kinesin and dynein are microtubule motor proteins involved in anterograde and retrograde trafficking respectively. A cross‐sectional view of a microtubule is shown to highlight its hollow lumen (created using biorender.com). B, A smooth muscle cell isolated from a rat mesenteric artery, stained for α tubulin and imaged with structured illuminated microscopy as per.^72^ This image shows that an extensive microtubule network exists in vascular smooth muscle cells

Microtubules form part of the cytoskeleton that is important for cell division; however, there is a growing appreciation of their importance in modulating signal transduction for a wide variety of extracellular ligands, as well as being a scaffold on which membrane proteins and proteins important for cell motility are transported.^57^, ^58^ By orchestrating the movement of proteins in and out of the cell membrane, microtubules can regulate cellular excitability and function.^59^ Microtubules are polymers of α‐ and β‐tubulin subunits associated laterally to form hollow tubes, which have the ability to dynamically grow (polymerization) and shrink (depolymerization) (see Figure 1).^60^, ^61^ This process is dependent on guanosine triphosphate (GTP), which stabilizes the polymerized state.^62^ After hydrolysis of GTP to guanosine diphosphate (GDP) at the growing end, depolymerization occurs and the microtubules disassemble.^62^, ^63^, ^64^ Microtubules are polar structures that harbour two distinct ends—the plus and minus ends—with the plus ends directed primarily towards the cell membrane and minus ends localized towards the cell centre. Microtubule organization within the cell is tightly controlled by a large number of microtubule‐associated proteins (MAPs) that promote or suppress dynamic behaviour at both of these ends.^60^ There is a long list of known MAPs, including various end‐binding (EB) proteins that localize to the plus end, as well as MAPs, such as MAP4, that localize along the lattice to promote microtubule stabilization.^65^ Increased binding of MAPs, such as MAP4, can prevent the movement of motor proteins (kinesin and dynein) along the microtubule.^60^, ^66^ Motor proteins are used in all eukaryotic cells to transport a variety of cargos, including membrane‐bounded organelles, mRNAs and proteins, along the “road network” created by microtubules. Dynein and kinesins are the two main classes of cargo‐transporting motors that utilize the microtubule network. Most kinesin family members transport cargoes towards the plus end, that is, the cell membrane,^67^ and dynein transports cargoes towards the minus end, that is, away from the cell membrane.^68^ Several mutations in microtubule‐based motors have been linked directly to neurological diseases.^58^

Post‐translational modifications of microtubules can have a dramatic effect on microtubule behaviour.^69^ Post‐translational modifications of microtubules include acetylation, phosphorylation and ubiquitination, most of which occur on the polymerized tubulin heterodimers. Importantly, there is growing recognition that post‐translational modifications of microtubules are involved in the pathogenesis of several diseases, for instance, acetylation has been implicated in neurodegenerative diseases such as Huntington's disease and Parkinson's disease.^70^

Although the microtubule network has been well studied in several cell types, particularly neurones, few studies have investigated its functional impact on smooth muscle contractility and arterial tone, even though an extensive network exists in these cells^71^, ^72^ (Figure 1).

## 4 THE MICROTUBULE NETWORK AND SMOOTH MUSCLE CONTRACTILITY

Research into the smooth muscle microtubule network has focused on its role in regulating smooth muscle cell migration.^73^ During migration, microtubules undergo polarization to control the signalling and trafficking of multiple proteins that regulate lamellipodial formation and focal adhesion dynamics. Although the role of the microtubule network in smooth muscle cell migration is well described (reviewed in detail by Ref.^74^), there have been few studies investigating the impact of microtubules on vascular smooth muscle contractility.

In order to study the role of the microtubule network in arteries, most studies have used microtubule depolymerizing agents, such as colchicine, nocodazole and demecolcine. All these compounds can bind to tubulin to prevent the polymerization of tubulin dimers, which promotes depolymerization of the microtubular structure. Colchicine is best known clinically for its anti‐inflammatory effects (elicited through the disruption of microtubules in pro‐inflammatory cells) making it an effective treatment for gout, as well as Familial Mediterranean Disease and, occasionally, for pericarditis.^75^ Colchicine binds to tubulin with high affinity resulting in a curved tubulin dimer, which prevents microtubule assembly.^76^ Demecolcine (also known as colcemid) is a structural analogue of colchicine and is able to bind to tubulin much faster than colchicine (equilibrium is attained within 45 minutes for demecolcine versus ~2 hours for colchicine), but is also more readily dissociated from tubulin when compared to colchicine.^77^ Nocodazole also interferes with the polymerization of microtubules and can depolymerize non‐acetylated tubulin, but interestingly does not affect polymerized acetylated microtubules.^78^

The first study to investigate the role of the microtubule network on arterial contractions showed that microtubule disruption by colchicine attenuated contractions to angiotensin and noradrenaline in isolated rabbit aortic strips,^79^ with no effect on basal tone. Conversely, several studies have found that microtubule disruption with colchicine, nocodazole or demecolcine can cause a contraction of arterial preparations, including porcine coronary arteries,^80^ rat aorta,^81^, ^82^, ^83^ cremaster^71^, ^84^ and skeletal muscle arteries,^85^ as well as enhancing contractions to noradrenaline.^86^ In all these studies, the constriction elicited by microtubule disruption has been highly variable. For example, the study by Zhang et al, which investigated the effects of colchicine and nocodazole on aortic tension from rest, only observed a small increase in tension (4%‐6% of the KCl contraction).^81^ In comparison, the study by Chitaley and Webb depolymerized the microtubule network in rat aortic rings with exposure to cold and stabilized this state by treatment with nocodazole or colchicine, which upon rewarming of vessels to 37°c, elicited a contraction twice as large in the colchicine and nocodazole‐treated vessels than DMSO‐treated vessels.^82^ Coronary artery rings from porcine hearts displayed isometric contractions to nocodazole that were only 1%‐2% of the maximal KCl contraction.^80^ Interestingly, this study found nocodazole enhanced contractions to 10 mM KCl by ~40%, but had no effect on 50 mM KCl contractions.^80^ In rat cremaster arteries mounted in a pressure myograph, colchicine, nocodazole and demecolcine all enhanced myogenic constrictions by ~30%, relative to basal tone.^71^ In this study, the increased tone was not accompanied by a change in intracellular Ca^2+^,^71^ whereas in porcine coronary arteries, nocodazole increased intracellular Ca^2+^ concentration by ∼15% of the maximal KCl‐stimulated increase in parallel with force, suggesting microtubule disruption could modulate Ca^2+^ signal transduction in these arteries.^80^ Moreover, pressurized rat skeletal muscle arterioles treated with demecolcine or vinblastine showed 20% more myogenic constriction than untreated vessels, as well as enhancing the contractions to noradrenaline.^85^ Ca^2+^‐dependent and independent constrictions were also enhanced in rat pulmonary artery preparations following in vivo administration of vinblastine.^87^ Unlike colchicine and nocodazole, vinblastine prevents tubulin polymerization by binding at the interface between two αβ–tubulin heterodimers, but rather than causing depolymerization, vinblastine stabilizes the microtubule plus end.^88^ Thus, vinblastine can inhibit the dynamic instability and growth of microtubules but does not completely remove the network. This raises the interesting possibility that the dynamic instability of the microtubule network is crucial for controlling certain contractile pathways in vascular smooth muscle.

Several of these studies found that Rho kinase activity was increased by microtubule disruption and the Rho kinase inhibitor, Y‐27632, prevented contractions in vessels caused by microtubule disruption.^81^, ^82^, ^85^ Rho kinase is involved in the Ca^2+^ sensitization pathway in smooth muscle. During a sustained contraction, the intracellular Ca^2+^ concentration decreases from its peak value; however, the contraction is still maintained.^89^ This mechanism is termed “Ca^2+^ sensitization”, which produces an increase in contractile force for a given concentration of intracellular calcium and allows a contraction to be maintained even at low concentrations of intracellular calcium. In brief, following myosin light chain kinase (MLCK) phosphorylation leading to the onset of smooth muscle contraction, myosin LC20 is dephosphorylated by myosin light chain phosphatase (MLCP) in order to terminate the contraction. Although phosphorylation of LC20 by MLCK is the main signalling event initiating smooth muscle cell contraction, inhibition of MLCP‐mediated dephosphorylation of LC20 is required for Ca^2+^ sensitization to produce a sustained contraction.^90^ It is now recognized that Ca^2+^ sensitization, because of MLCP inhibition, occurs predominantly through signalling pathways involving the Ras homolog gene family, member A (RhoA)/Rho‐associated kinase (ROK) and protein kinase C (PKC).^91^ Upon ligand binding to the G12/13‐coupled receptor, guanine nucleotide exchange factors (GEFs) are activated, which facilitate the switch between RhoA bound to GDP to RhoA bound to GTP. RhoA‐GTP is the active form of RhoA, which in turn activates ROK.^90^ ROK can inhibit MLCP by directly phosphorylating the myosin phosphatase target (MYPT) 1 subunit, and by phosphorylating the PKC‐potentiated protein phosphatase 1 inhibitor (CPI‐17), which also phosphorylates MYPT1.^92^, ^93^ In addition, Gq/11 protein‐coupled receptor occupancy leads to PKC activation. PKC also phosphorylates the MYPT1 subunit of MLCP,^92^, ^94^ thus inhibiting MLCP‐mediated dephosphorylation of LC20.^90^

Although microtubule depolymerization induces an increase in RhoA activity, the precise mechanisms underlying this increase in RhoA activity are unclear in vascular smooth muscle. It is also not clear whether the microtubule network is able to regulate RhoA activity in vascular smooth muscle, thereby controlling the Ca^2+^ sensitization in a given myocyte. One possible mechanism for the increased RhoA activity in vascular smooth muscle following microtubule disruption has been identified in cultured striatal neuronal cells, expressing mutant huntingtin.^95^ In these cells, colchicine caused the release and activation of the microtubule‐associated Rho activator, GEF‐H1, which increased RhoA‐ROK signalling.^95^ Investigating these mechanisms will provide important information regarding the involvement of the microtubule network in the Ca^2+^ sensitization pathway in smooth muscle.

More recently, microtubule depolymerization, with nocodazole, disrupted the tight interactions between the sarcoplasmic reticulum and the plasma membrane in the smooth muscle cells. Nocodazole increased the distance between the ryanodine receptors found on the sarcoplasmic reticulum and BK_Ca_ channels in the plasma membrane, which led to a reduced BK_Ca_ channel activity. By reducing the ability of the Ca^2+^ sparks to activate the BK_Ca_ channels, nocodazole treatment was associated with increased pressure‐induced constriction of the cerebral resistance arteries.^96^

Although these studies show that disruption of the microtubule network has the potential to affect arterial tone by influencing several mechanisms controlling intracellular Ca^2+^ and Ca^2+^‐sensitization pathways, no studies had investigated the microtubule‐dependent trafficking of membrane proteins that influence contractility in vascular smooth muscle.

## 5 MICROTUBULE TRAFFICKING OF KV CHANNELS

The microtubule network is responsible for transporting a wide variety of proteins throughout the cell, using kinesin and dynein to control the direction of movement. Kv channels play important roles in several cellular systems, predominantly contributing to changes in the electrical activity of the cell. Thus, the trafficking mechanisms determining the membrane expression of Kv channels are important regulators of cellular excitability. The microtubule network can traffic specific α‐subunit proteins of Kv channels in various cell systems. Most investigations have focused on the Kv1 channel family and, more specifically, the Kv1.5 channel. The first evidence of the microtubule network trafficking Kv1.5 channels came in a study from 2001, which showed that the channel resided in caveolae of mouse L‐cells and was internalized following microtubule disruption by demecolcine, along with the caveolae, whereas the cell surface localization of Kv2.1 channels was not affected by demecolcine treatment in L‐cells.^97^ A more detailed analysis of Kv1.5 channel transport by a microtubule‐dependent trafficking mechanism came in 2005 when Fedida and colleagues showed that nocodazole treatment doubled the Kv1.5 current in HEK293B cells and doubled the sustained current attributed to Kv1.5 in rat atrial myocytes.^98^ These electrophysiological effects were supported by immunostaining of rat atrial myocytes, which showed increased Kv1.5 channel membrane expression.^98^ This study further found that the Kv1.5 current increased in HEK293B cells after co‐expression of p50/dynamitin to inhibit dynein, as well as treatment with a dynamin inhibitory peptide, which blocks endocytosis. Moreover, the authors performed co‐immunoprecipitation assays to determine that Kv1.5 proteins interact with dynein in HEK293B cells and ventricular myocytes.^98^ Thus, the retrograde trafficking of Kv1.5 channel is controlled by dynein and the microtubule network. Anterograde trafficking of Kv1.5 is also performed by the microtubule network and is dependent on kinesin transport (Kif5B, specifically).^99^ In HEK293 cells and H9c2 cardiomyoblasts, Kv1.5 current densities were almost doubled when the Kif5b motor was overexpressed.^99^ Recently, colchicine treatment of rat atrial myocytes did not affect the Kv1.5 current suggesting that anterograde and retrograde trafficking were equally affected.^100^ Further dissection of these pathways in atrial myocytes found that colchicine could both reduce membrane expression of the Kv1.5 channel by ~40% and inhibit endocytosis of the channel by ~37%.^100^ This study also showed an important role for clathrin vesicles in the microtubule‐dependent trafficking of Kv1.5. In control conditions, when the channel was located in clathrin vesicles, there was a strong association between the EB1 protein of the microtubule network and Kv1.5 proteins. Since dynein interacts indirectly with the EB1 protein and clathrin blockade mirrored the effects of colchicine, the authors suggest that the microtubule network and dynein are involved in the early stages of internalization and are responsible for the retrograde trafficking of the channel in clathrin vesicles.^100^

Kinesin isoform Kif5B regulates the transport of Kv1.1, 1.2, 1.3 and 1.4 in cultured cortical neurones or slices.^101^ The authors used a dominant‐negative variant of Kif5B, which blocked the normal axonal localization of these channels. The interaction of these Kv1 channels with Kif5B was controlled by their T1 domain, which is known to be important for the targeting of the channel to the axon.^101^ The authors did not establish whether the interaction of Kif5B with the T1 domain was direct or indirect, nor what caused the targeting of Kif5B to the axon. Thus it is likely, several other proteins are involved in this transport process. Kv1.2 targeting to the axon was found to be dependent on the ancillary subunit Kvβ2, which associates with kinesin II and the EB1, and directs the trafficking of Kv1.2.^102^ This process was later shown to be regulated by cyclin‐dependent kinase‐mediated phosphorylation‐dependent binding of Kvβ2 to EB1.^103^

Microtubule‐dependent trafficking of other Kv channels has also been shown, but the evidence for this is sparse. In rat ventricular myocytes, nocodazole enhanced A‐type K^+^ currents by ~60%, but reduced inward‐rectifier K^+^ currents by ~33%. Thus, nocodazole led to a reduced action potential duration in the ventricular myocytes. Further investigation showed nocodazole and inhibition of dynein by p50 overexpression increased the membrane protein levels and current of Kv4.2 in HEK293B cells.^104^ Expression levels of Kv2.1 and Kv3.1 were also increased by p50 co‐expression in HEK293B cells in this study. Additionally, paclitaxel, the microtubule stabilizer, inhibited Kv2.1 channel currents in H9c2 cells.^105^ In this study, the authors only detected a change in the Kv2.1 current with 1 and 10 µmol/L of paclitaxel, which is far above its normal IC_50_.^106^ Although the authors suggest paclitaxel is an open channel blocker of Kv2.1 channels, the possibility that paclitaxel increased the retrograde trafficking of Kv2.1, which could contribute to the observed time‐dependent paclitaxel inhibition of the Kv2.1 current, cannot be ruled out since these high doses of paclitaxel were only applied for 5 mins. Longer exposure to lower concentrations of paclitaxel is required to establish whether stabilization of the microtubule network affects Kv2.1 trafficking.

In 2008, a link between Kv7 channels and the microtubule network was first described. Nicolas et al showed that the enhancing effect of protein kinase A on the cardiac slow delayed rectifier K^+^ current (I_Ks;_ formed by the co‐assembly of Kv7.1 and KCNE1) was reduced after microtubule disruption by colchicine in COS‐7 cells and guinea pig cardiac myocytes, whereas no effect was observed on basal the I_Ks_. Since Kv7.1 channel membrane expression was not affected following microtubule disruption, the authors concluded that the microtubule network regulated the coupling of PKA‐dependent Kv7.1 phosphorylation and activation of the I_Ks_.^107^ These findings implicate the microtubule network in the normal signalling pathways that are crucial for regulating the cardiac action potential. PKA can affect a variety of channels in cardiac myocytes, yet no further study has investigated whether the microtubule network is involved in the cell signalling of PKA in cardiac myocytes.

Although these studies show that microtubules can traffic Kv channels, no study had investigated the trafficking of vascular smooth muscle Kv channels by the microtubule network.

## 6 THE MICROTUBULE NETWORK IS RESPONSIBLE FOR RETROGRADE TRAFFICKING OF Kv7.4 CHANNELS IN VASCULAR SMOOTH MUSCLE

**FIGURE 2:**
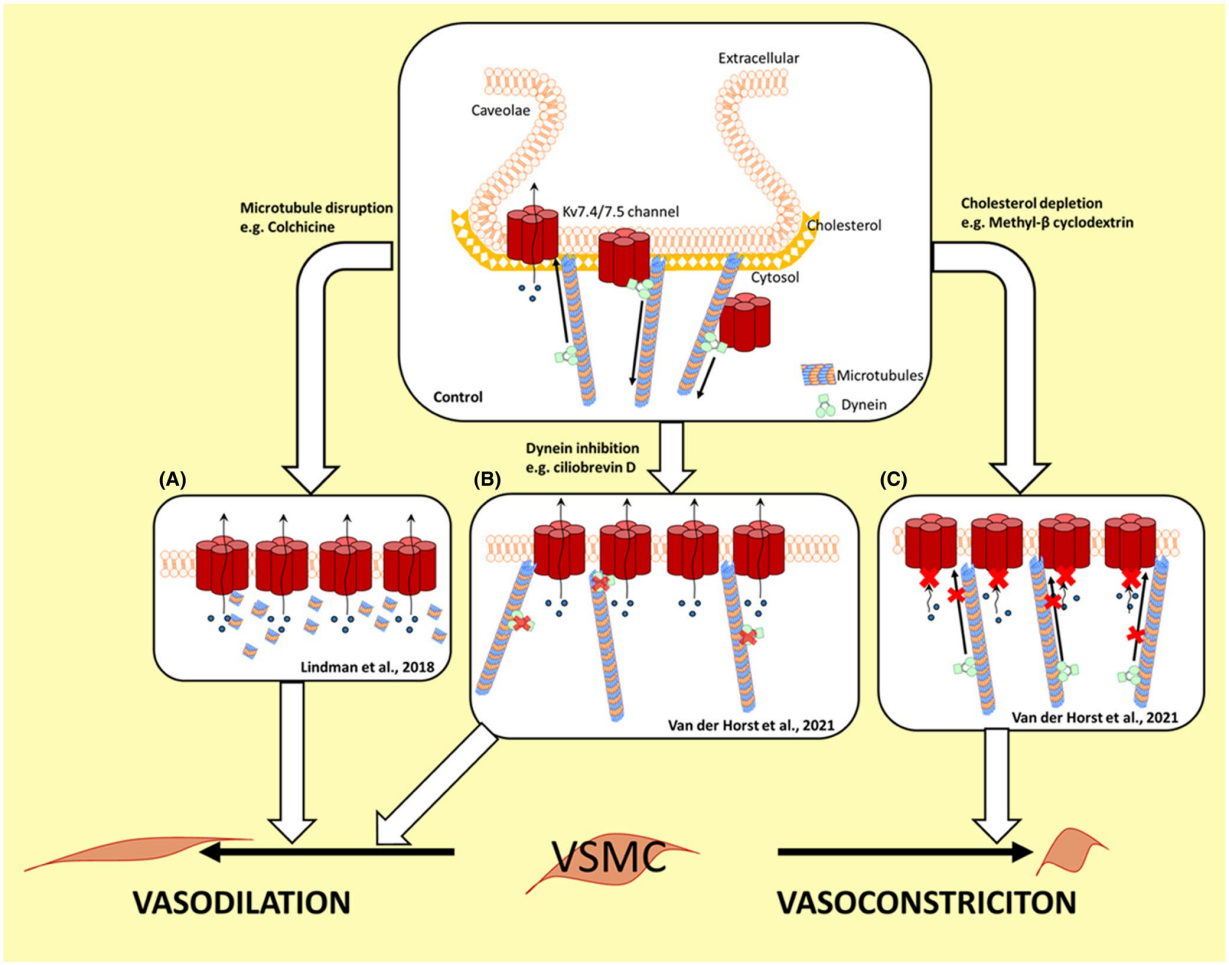
Schematic summarizing the findings in Lindman et al^72^ and van der Horst et al.^109^ Under control conditions, the microtubule network and dynein facilitates the removal of Kv7.4 channels from the membrane VSMCs, which is dependent on cholesterol‐rich caveolae. A, When the microtubule network was disrupted, the number of Kv7.4 channels in the membrane increased, which promoted vasorelaxations. B, The Kv7.4 protein contains a dynein‐binding motif in its intracellular C‐terminus allowing dynein to attach. Ciliobrevin D prevents dynein from using ATP, thereby stopping dynein movement along the microtubule network. In the presence of ciliobrevin D, Kv7.4 protein expression increased in the membrane of vascular smooth muscle cells (VSMCs), which enhanced vasorelaxations to Kv7 activators. C, Dynein clusters to cholesterol‐rich microdomains and Kv7.4 channels reside in cholesterol‐rich caveolae. Depletion of cholesterol reduces the ability of dynein to interact with the Kv7.4 protein; therefore, the number of Kv7.4 channels in the membrane increases. However, without cholesterol, the Kv7 channels are unable to function properly, leading reduced relaxations from Kv7 channel activators. Although dynein can bind to the Kv7.4 C‐terminus, it should be noted that it is still unclear whether dynein internalizes the whole caveolae vesicle or a subpart containing the Kv7.4 protein in this process

Given the importance of Kv7 channels in the vasculature, our laboratory investigated whether the microtubule network was involved in the trafficking of Kv7 channels to or from the cell membrane.^72^ Our initial experiments pre‐treated segments of rat mesenteric and renal artery, under isometric tension, for 1 hour with colchicine and nocodazole to depolymerize the microtubule network. In these arteries, we assessed relaxations to the Kv7.2‐7.5 channel activator S‐1 and found that both microtubule disruptors enhanced the relaxations. In line with Kv7 channels contributing to β adrenoceptor‐mediated relaxations, we also found that colchicine and nocodazole improved isoprenaline‐mediated relaxations. The improved relaxations were attenuated by the Kv7 channel blocker, XE991, suggesting the increased Kv7 channel function elicited by microtubule disruption partially underlies improved β adrenoceptor relaxations.^72^ Microtubule disruption has the potential to disturb multiple cell signalling pathways in vascular smooth muscle, including calcium handling, as described above. To show that colchicine improved relaxations to isoprenaline were through increased Kv7 channel function, specifically, we measured changes in membrane potential and intracellular calcium in arterial segments, while recording changes in tension, simultaneously. These experiments showed that a low concentration of isoprenaline, which had no effect on tension, membrane potential or cytosolic calcium in untreated mesenteric artery segments, produced a hyperpolarization, decreased cytosolic calcium and relaxed arteries in the presence of colchicine. All of these effects were inhibited by 10 µmol/L of the Kv7 channel blocker, XE991. We performed structured illumination microscopy to determine whether the increased Kv7 channel function in arteries treated with colchicine was because of an increase in the number of channels in the cell membrane. These experiments showed that more Kv7.4 protein was present in the smooth muscle cell membrane after colchicine treatment, with no change in the overall Kv7.4 protein expression in these arteries.^72^ Figure 2A summarizes these findings.

Following this study, we determined how the microtubule network trafficked Kv7.4 channels. Since disruption of the microtubule network led to increased Kv7.4 channel expression in the membrane, we hypothesized that under normal conditions microtubules assisted in the removal of Kv7.4 channels from the membrane. Of the two main types of microtubule motor protein (kinesin and dynein), retrograde transport is controlled by dynein. In our study published recently, we identified two dynein recognition motifs in the intracellular C‐terminus of the Kv7.4 protein between the so‐called C and D helices.^108^ We mutated a glutamine (580) in one of these recognition motifs to an alanine and found that the Kv7.4 current was increased in HEK293B cells compared to the wild‐type channel. Co‐expression of dynamitin/p50, which inhibits dynein function, increased wild‐type Kv7.4 currents in HEK293B cells, but had no effect on the mutant Kv7.4 currents.^109^ Interestingly, this dynein recognition motif is conserved in the other Kv7 channel members, suggesting that retrograde trafficking by dynein could be an important feature of other Kv7 channel isoforms, which are known to be important in multiple cells throughout the body.^23^

After showing dynein was expressed in rat mesenteric arteries, we used co‐immunoprecipitation and proximity ligation assays to show that dynein co‐localized with Kv7.4 proteins in mesenteric artery smooth muscle cells. To investigate the functional impact of dynein on Kv7 channel function in arteries, we used a recently discovered inhibitor of dynein, ciliobrevin D.^110^ Ciliobrevins are a group of small molecules, which target the dynein ATPase to act as an ATP competitor. This action inhibits the motor activity of dynein without disturbing dynein binding to microtubules.^111^ The projected IC_50_ of ciliobrevin D to inhibit dynein movement is between 10 and 40 µmol/L^110^, ^111^; therefore, to avoid off‐target effects, we used 10 µmol/L in our study. Application of 10 µmol/L ciliobrevin D to rat resistance mesenteric arteries under isometric tension in a wire myograph enhanced vasorelaxations to the Kv7 channel activators S‐1 and NS15370^109^ (see summary schematic in Figure 2B). This effect was dependent on Kv7.4 channels since morpholino knockdown of Kv7.4 in mesenteric artery segments prevented ciliobrevin D from enhancing the S‐1‐dependent relaxations.

Further investigation using mass spectrometry, co‐immunoprecipitation assays and proximity ligation assays showed that the dynein targeting of Kv7.4 was dependent on cholesterol‐rich caveolae.^109^ Disruption of the caveolae with 5 mmol/L methyl‐β cyclodextrin reduced dynein's interaction with Kv7.4 and caused an increase in the membrane expression of the channel^109^ (see summary schematic in Figure 2C). Our data showed that cholesterol is an integral component of this dynein‐dependent trafficking mechanism, which is in line with previous evidence that dynein clusters to cholesterol‐rich microdomains.^112^, ^113^, ^114^, ^115^ Although membrane expression of Kv7.4 protein increased, relaxations to Kv7 activators were attenuated by methyl‐β cyclodextrin treatment, which also inhibited the effect of ciliobrevin on arterial relaxations to the Kv7 activators ^109^ (see summary schematic in Figure 2C). Further analysis revealed that Kv7.4 currents in oocytes were inhibited by cholesterol depletion.^109^ These data suggest that the channel requires cholesterol to function properly, which has also been shown for other Kv7 channels.^116^ We do not know the precise mechanism by which Kv7.4 or caveolae vesicles are internalized and trafficked by dynein. Whether the Kv7.4 C‐terminal dynein recognition motif acts as a dynein binding site on an endocytosed caveolae vesicle remains to be determined. Furthermore, other ion channels and receptors residing in caveolae should be investigated to determine whether all caveolae undergo dynein‐dependent retrograde trafficking. The regulatory role of cholesterol in this process has also not been explored fully. For example, dynein tethering and the minus‐end transport of certain vesicles is dependent on a cholesterol‐sensing switch, oxysterol‐binding protein‐related protein 1L (ORP1L) as well as the Rab GTPase, Rab 7.^114^, ^115^, ^117^ Investigating these proteins and their associations with caveolae and Kv7.4 will further our understanding into the regulation of this dynein trafficking mechanism by cholesterol.

## 7 FUTURE PERSPECTIVES

It is important to note that these data only scratch the surface of potential physiological roles of the microtubule network in arterial smooth muscle. The trafficking of several membrane proteins is likely to be along the road network created by microtubules and the associated motor proteins. This potential is highlighted by our finding that dynein interacts with caveolae. Many different membrane proteins are known to localize in these cholesterol‐rich membrane invaginations to create important microdomains. If dynein is responsible for the retrograde trafficking of caveolae vesicles, then the trafficking of many other ion channels, receptors and signalling complexes will be dependent on the microtubule network.

In hypertension, Kv7.4 channel expression is decreased in arterial smooth muscle and function is attenuated. Our understanding of what drives these changes in hypertension is limited despite offering a new avenue to combat vascular disease. Previously, E3 ubiquitin ligase CHIP (C terminus of heat shock protein 70 (Hsp70)‐interacting protein) was found to promote ubiquitination and proteosomal degradation of Kv7.4 channels in arterial smooth muscle cells, in a mechanism that is up‐regulated by angiotensin II.^52^ CHIP‐induced ubiquitination can lead to association with dynein for retrograde transport and degradation by the aggresome.^118^ Interestingly, we found unique peptides for Hsp70 in dynein immunoprecipitate from rat mesenteric arteries by mass spectrometry analysis, although these data are not published and need to be verified. This raises an intriguing possibility that CHIP, dynein and the microtubule network regulate the retrograde trafficking and degradation of Kv7.4 channels, in a mechanism that is augmented by angiotensin II, thereby underling the reduced Kv7.4 expression in arteries from hypertensive arteries.

Alterations in the trafficking ability of the microtubule network are implicated in several diseases including Huntington's disease,^119^ Parkinson's disease^120^ and heart failure^121^, ^122^; however, the pathophysiological role of the microtubule network has not been studied in hypertensive arteries. Could changes in microtubule network dynamics contribute to the pathological changes observed in arteries from hypertensive animals and patients? Future research will investigate changes in the microtubule network in arteries from hypertensive patients and animals to determine its contribution to the disease.

## 8 CONCLUSION

The microtubule network is a road network within cells that has been described for decades.^123^, ^124^ In the mid‐1980s, the first motors were found to be travelling along the microtubule network in order to transport cargo to different parts of the cell.^125^, ^126^ However, it is only relatively recently that problems with the microtubule network, or dysfunction of the motors, have been implicated in different diseases. The scope and impact of the microtubule network and its motors on the trafficking of various proteins in different cell types is now being realized, but there is still much we do not know. Our work has unveiled a novel trafficking role involving the microtubule network in vascular smooth muscle. Importantly, the implications of this trafficking mechanism are still to be determined in vascular diseases such as hypertension.

## CONFLICT OF INTEREST

The author declares no conflict of interest.
